# Nicotinamide Mononucleotide Restores the Meiotic Competency of Porcine Oocytes Exposed to Ethylene Glycol Butyl Ether

**DOI:** 10.3389/fcell.2021.628580

**Published:** 2021-02-02

**Authors:** Yilong Miao, Xinyu Li, Xiaoyan Shi, Qian Gao, Jingyue Chen, Rui Wang, Yong Fan, Bo Xiong

**Affiliations:** ^1^College of Animal Science and Technology, Nanjing Agricultural University, Nanjing, China; ^2^Key Laboratory for Major Obstetric Diseases of Guangdong Province, Department of Obstetrics and Gynecology, The Third Affiliated Hospital of Guangzhou Medical University, Guangzhou, China; ^3^College of Veterinary Medicine, Nanjing Agricultural University, Nanjing, China

**Keywords:** EGBE, NMN, oocyte quality, cytoskeleton, mitochondrial function

## Abstract

Ethylene glycol butyl ether (EGBE), a type of glycol ethers, is a common chemical used in both industrial and household products. Increasing animal studies have indicated that it produces reproductive problems, such as testicular damage, reduced female fertility, death of embryos, and birth defects. However, how it influences the female germ cells has not yet determined. Here, we found that EGBE exposure resulted in the defective porcine oocyte maturation via disruption of cytoskeleton dynamics, showing the abnormal spindle assembly, chromosome alignment, and actin organization. Meanwhile, EGBE exposure perturbed the mitochondrial distribution and function, leading to the accumulation of reactive oxygen species (ROS) and generation of DNA damage and apoptosis. Of note, nicotinamide mononucleotide (NMN) supplementation rescued the meiotic defects caused by EGBE exposure via restoring NAD^+^ level and mitochondrial function and thus eliminating the excessive ROS. Taken together, our observations illustrate that NMN supplementation is an effective strategy to protect oocyte quality against environmental pollutant-induced deterioration, contributing to improve the animal and human fertility.

## Introduction

The glycol ethers (GEs) consists of a class of organic solvents widely applied in the household and industrial products (Browning and Curry, 1994). At present, the chemical industry has synthesized more than 30 different GEs (Multigner et al., 2005), and ethylene glycol monobutyl ether (EGBE), ethylene glycol monoethyl ether (EGEE), and ethylene glycol monomethyl ether (EGME) are the most widely consumed products (Fort et al., 2018). Due to their potential toxicity and widespread availability, it has attracted more attentions. A previous study on the EGME-exposed female workers shows the extended menstrual cycles and the time to pregnancy (Hsieh et al., 2005). Furthermore, *in vitro* investigation of meiotic resumption in Xenopus oocytes has indicated that EGME influences the critical process of oocyte maturation prior to fertilization (Fort et al., 2002). EGEE is soluble in water or other solvents and absorbed through the skin of the body. EGEE in the body is then converted to ethoxy acetaldehyde (EAA) and ethoxy acetic acid (EAA), two toxic metabolites, by ethanol dehydrogenase and aldehyde dehydrogenase (Cheever et al., 1984; Kennedy et al., 1993). Recent studies have found that EGEE exposure causes the health risks including the damage to the liver, blood, and reproductive systems (Welsch, 2005; Scofield et al., 2006; Wang et al., 2006). Repeated exposure to EGEE causes testicular atrophy, reduced sperm count, and sperm motility retardation (Melnick, 1984; Wang et al., 2006, 2007). EGBE is rapidly absorbed through dermal, pulmonary, and gastrointestinal routes (Bauer et al., 1992). The acute exposure to EGBE not only results in renal, neurologic, hematologic, and metabolic disorders but also leads to acute severe respiratory failure (Bauer et al., 1992). The reproductive toxicity of EGBE has been also reported in female mice by showing the subfertility (Cicolella, 2006). Nevertheless, the underlying mechanisms concerning how EGBE impacts the female fertility and the quality of oocytes remain largely unknown.

Nicotinamide adenine dinucleotide (NAD^+^) is a coenzyme for multiple redox reactions involved in glycolysis, oxidative phosphorylation and tricarboxylic acid cycle in cells (Camacho-Pereira et al., 2016). In particular, NAD^+^ has effects on a variety of cellular endpoints directly or indirectly as a rate-limiting substrate for sirtuin proteins, the critical enzymes to regulate the mitochondrial homeostasis and pro-survival pathways (Anastasiou and Krek, 2006; Csiszar et al., 2008, 2009a,b; Das et al., 2018). It has been reported that decrease of intracellular NAD^+^ changes the NAD^+^/SIRT1 pathway and compromises the mitochondrial function, thereby leading to the ROS accumulation, DNA damage and apoptosis in neurons (Croteau et al., 2017). Notably, NMN, a key precursor to NAD^+^, has been implicated in replenishing NAD^+^ levels and restoring the expression of genes related to circadian rhythm, inflammatory response, and oxidative stress by activation of Sirt1 in both HFD-induced and aged mice (Yoshino et al., 2011).

In the present report, we investigated the potential toxicity of EGBE on the oocyte meiotic progression and cytoskeleton dynamics in pigs. We also validated the beneficial impact of NMN supplementation on the developmental competency of EGBE-exposed oocytes.

## Materials and Methods

### Antibodies

Mouse monoclonal α-tubulin-FITC (fluorescein isothiocyanate) and acetyl-α-tubulin (Lys40) antibodies were obtained from Sigma-Aldrich (St Louis, MO, United States; Cat# F2168, ABT241); rabbit monoclonal GAPDH and Phospho-Histone H2A.X (Ser139) antibodies were obtained from Cell Signaling Technology (Danvers, MA, United States; Cat# 2118, 9718).

### Collection of Porcine Oocytes

Porcine ovaries were obtained from a local abattoir and transported to the laboratory in a physiological saline containing streptomycin sulfate and penicillin G within 2 h after slaughtering. Cumulus-oocyte complexes (COCs) were aspirated from the follicles using a disposable syringe. COCs with a compact cumulus cells were selected for *in vitro* maturation (IVM). The maturation medium is TCM-199 (Thermo Fisher Scientific, Waltham, MA, United States; Cat# 11150059) supplemented with 10% porcine follicular fluid, 5 μg/mL insulin, 10 ng/mL EGF, 0.6 mM cysteine, 0.2 mM pyruvate, 25 μg/mL kanamycin, and 10 IU/mL of each eCG and hCG. 20 germinal vesicle (GV) COCs were cultured in a drop of 100 μL maturation medium covered with mineral oil at 38.5°C, 5% CO_2_ for 26–28 h to metaphase I stage and for 42–44 h to metaphase II stage.

### EGBE and NMN Treatment

Ethylene glycol butyl ether (EGBE) was obtained from Sigma-Aldrich (Cat# 53071) and dissolved in the water to 500 mM for a stock solution, which was further diluted with maturation medium to a working concentration of 50, 100, or 250 μM, respectively. Nicotinamide mononucleotide (NMN; GeneHarbor Biotech, Hong Kong, China) was dissolved in the water to 1 M for a stock solution and diluted with maturation medium to a working concentration of 100 nM, 10 μM, and 1 mM, respectively.

### Measurement of NAD^+^ Levels

NAD^+^ levels were measured with a NAD/NADH Quantitation Kit (Sigma-Aldrich; Cat# MAK037) according to the manufacturer’s instruction. In brief, 120 oocytes were harvested for total NAD^+^ extraction and quantification based on the procedure described by Pantazi et al. (2015). The NAD^+^ concentration was calculated by subtracting the NADH values from NAD_to__tal_ (NAD^+^ and NADH). NAD_to__tal_ and NADH levels were quantified in a colorimetric assay at 450 nm using iMark^TM^ Microplate Absorbance Reader (Bio-Rad, Hercules, CA, United States).

### Fluorescence Staining and Confocal Microscopy

Denuded oocytes (DOs) were obtained by transferring COCs in 300 μL of 10 mg/mL hyaluronidase solution for 15 min. Then DOs were incubated in the fixation solution (4% paraformaldehyde/PBS) for 30 min, in the permeabilization solution (1% Triton X-100/PBS) for 1 h, and in the blocking solution (1% BSA-supplemented PBS) for 1 h at room temperature (RT), followed by incubation with α-tubulin-FITC antibody (1:200), acetyl-α-tubulin antibody (1:100), γH2A.X antibody (1:100) or phalloidin-TRITC (1:100; Sigma-Aldrich; Cat# P1951) overnight at 4°C. After washes in PBST, oocytes were incubated with the corresponding secondary antibodies for 1 h and counterstained with 10 μg/mL Hoechst 33342 or propidium iodide (PI) for 10 min at RT. In addition, oocytes were stained at 38.5°C for 30 min with 500 nM MitoTracker Red CMXRos (Thermo Fisher Scientific; Cat# M7512) for mitochondrion staining, with 2 μM MitoProbe JC-1 (Thermo Fisher Scientific; Cat# M34152) for mitochondrial membrane potential assessment, with 10 μM dichlorofluorescein diacetate (DCFHDA; Beyotime, Huangzhou, China; Cat# S0033S) for ROS staining, and with Annexin-V-FITC (1:10; Beyotime; Cat# C1062) for apoptosis assessment. Lastly, oocytes were mounted on the glass slides and imaged under a confocal microscope (LSM 700 META, Zeiss, Germany).

### Immunoblotting

Porcine oocytes were collected in the lysis buffer (4 × LDS sample buffer, Thermo Fisher Scientific; Cat# NP0007) with protease inhibitor and heated at 95°C for 5 min. Proteins were separated on 10% precast gels (Bis-Tris) and transferred to PVDF membranes. The blots were then incubated in the blocking buffer (5% low fat dry milk/TBST) for 1 h at RT and probed with acetyl-α-tubulin antibody (1:1,000) or GAPDH antibody (1:5,000) overnight at 4°C. After washes in TBST, the blots were incubated with the corresponding secondary antibodies for 1 h at RT. Chemiluminescence signals were acquired with ECL Plus (ThermoFisher Scientific; Cat# 32132) and protein bands were detected by Tanon-3900 Imaging System.

### Evaluation of Total ATP Content

Total ATP content in a pool of 20 oocytes was determined using the Bioluminescent Somatic Cell Assay Kit (Sigma-Aldrich; Cat# FLASC), following the procedure described by Combelles and Albertini (2003) and the manufacturer’s instruction. A 5-point standard curve (0, 0.1, 0.5, 1.0, 10, and 50 pmol of ATP) was generated in each assay and the ATP content was calculated by using the formula derived from the linear regression of the standard curve.

### RNA Extraction and Quantitative Real-Time PCR

Fifty oocytes were collected to extract the total RNA using RNeasy Mini Kit (Qiagen, Germantown, MD, United States; Cat# 74104) which was then reversed to cDNA with PrimeScript RT Master Mix (Takara, Kusatsu, Shiga, Japan; Cat# RR036A). Quantitative real-time PCR was performed using Real-Time PCR System (QuantStudio 7 Flex, Thermo Fisher Scientific) with SYBR Green PCR Master Mix (Thermo Fisher Scientific; Cat# 4344463). Data were normalized with *GAPDH* and the fold change was quantified by the comparative CT method. The primers were listed as follows:

*GSR* (F: ACAGTGGGACTCACAGAAGA/R: AGGTAGGAT GAATGGCAAC);

*GPX1* (F: CCAAGTTTATCACCTGGTCTCC/R: AGGCACT GCTAGGCTCCTG); *GPX4* (F: TGTGGTTTACGGATTCTGG/R: CCTTGGGCTGGACTTTCA);

*SOD1* (F: GGTCCTCACTTCAATCCTG/R: CTTCATTTC CACCTCTGC);

*SOD2* (F: TATCCGTCGGCGTCCAAG/R: GCGGCGTATCG CTCAGTT);

*GAPDH* (F: TGGGCTACACTGAGGACC/R: TACCAGGA AATGAGCTTGA).

### Statistical Analysis

Data from at least three independent replicates were designated as mean percentage or value (mean ± SEM). Differences between two groups were analyzed by paired-samples *t*-test using GraphPad Prism 6 statistical software. *P* < 0.05 was accepted to be significant.

## Results

### NMN Recovers the Meiotic Maturation of EGBE-Exposed Porcine Oocytes

**FIGURE 1:**
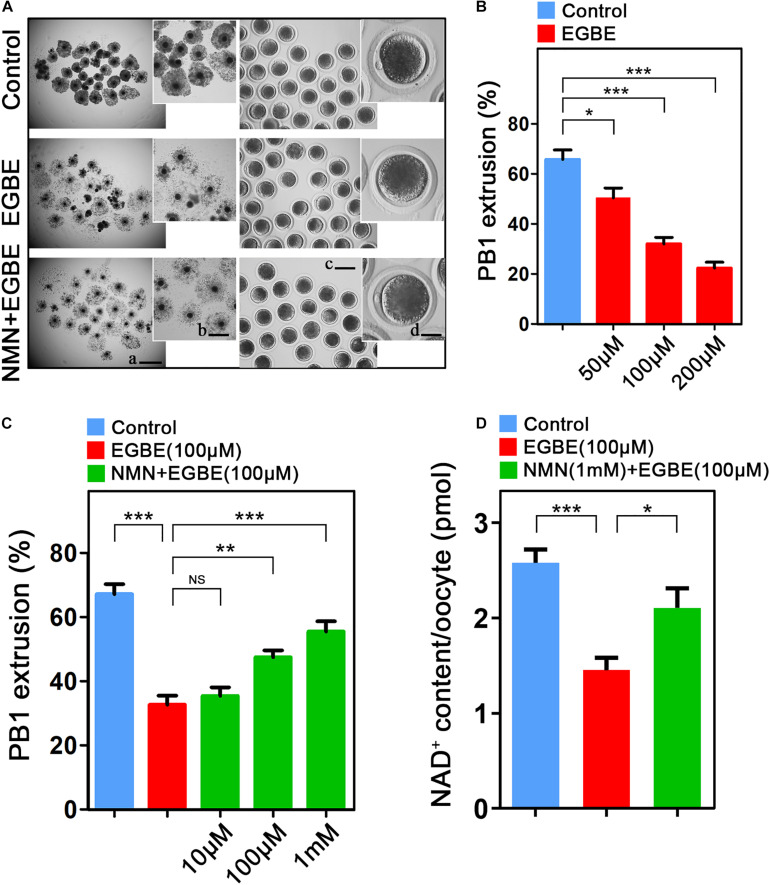
Effect of NMN supplementation on the porcine oocyte maturation after EGBE exposure. **(A)** Representative images of *in vitro* matured oocytes in control, EGBE-exposed, and NMN-supplemented groups. Polar body extrusion and cumulus cell expansion of oocytes were imaged by the confocal microscope. Scale bars: (a) 800 μm; (b) 400 μm; (c) 150 μm; (d) 30 μm. **(B)** The proportion of polar body extrusion was calculated in control and different concentrations of EGBE-exposed oocytes (50, 100, and 200 μM) after *in vitro* maturation. **(C)** The proportion of polar body extrusion was calculated in control, EGBE-exposed and different concentrations of NMN-supplemented oocytes (10, 100 μM, and 1 mM) after *in vitro* maturation. **(D)** NAD^+^ levels were measured in control, EGBE-exposed and NMN-supplemented oocytes. Data were expressed as mean percentage or value (mean ± SEM) of at least three independent experiments. *P < 0.05, **P < 0.01, ***P < 0.001, NS, no significance.

To examine the influence of EGBE on the meiotic progression of porcine oocytes, increasing concentrations of EGBE (50, 100, and 200 μM) were added to the *in vitro* maturation medium. We observed that EGBE exposure prominently led to the oocyte meiotic failure by showing the defective expansion of cumulus cells and the reduced proportion of oocytes that completed the maturation (Figure 1A). Quantification data revealed that exposure to different doses of EGBE all caused a decline in the polar body extrusion (PBE) after IVM for 44 h, and addition with 100 and 200 μM EGBE considerably lowered the rate of PBE from 67% in controls to 32 and 22%, respectively (66.7 ± 2.8%, *n* = 112, control vs. 32.1 ± 2.4%, *n* = 101, 100 μM EGBE, *P* < 0.001 vs. 22.3 ± 2.3%, *n* = 109, 200 μM EGBE, *P* < 0.001; Figure 1B). We then used 100 μM EGBE for subsequent studies. To test whether NMN can improve the defective oocyte development induced by EGBE exposure, we supplemented different doses of NMN (10, 100 μM, and 1 mM) with 100 μM EGBE in the maturation medium. As expected, 1 mM NMN increased the proportion of PBE in EGBE-exposed oocytes to the level indistinguishable from the controls (67.2 ± 2.1%, *n* = 117, control, *P* < 0.001 vs. 32.7 ± 2.8%, *n* = 120, EGBE vs. 55.5 ± 2.1%, *n* = 110, 1 mM NMN, *P* < 0.001; Figure 1C). We further detected the NAD^+^ levels after EGBE exposure and NMN supplementation in oocytes. The results displayed that NMN supplementation significantly elevated the NAD^+^ levels that were reduced in EGBE-exposed oocytes (2.58 ± 1.13, *n* = 120, control, *P* < 0.001 vs. 1.45 ± 1.55, *n* = 120, EGBE vs. 2.11 ± 1.24, *n* = 120, NMN, *P* < 0.05; Figure 1D). Altogether, these results indicate that NMN can recover the failure of porcine oocyte meiotic maturation induced by EGBE exposure via restoring the NAD^+^ level.

### NMN Restores the Spindle/Chromosome Structure and Microtubule Dynamics in EGBE-Exposed Porcine Oocytes

**FIGURE 2:**
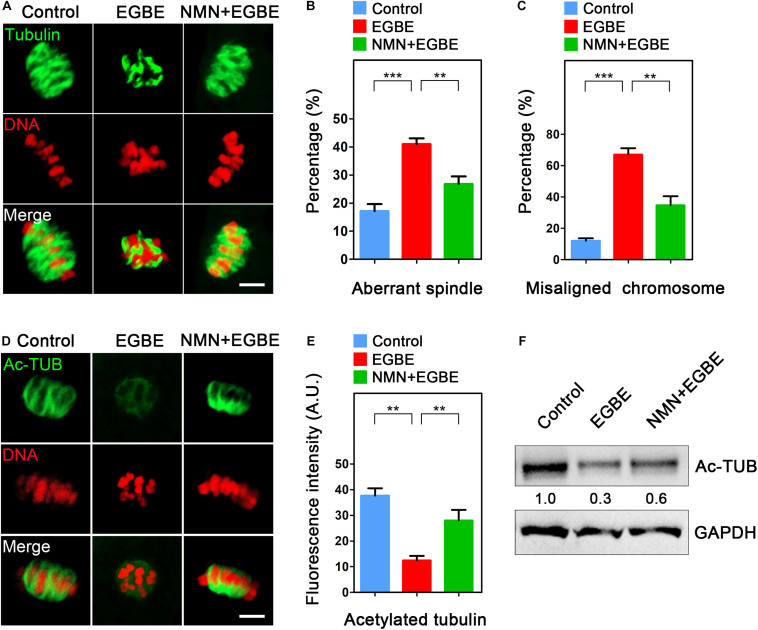
Effect of NMN supplementation on the spindle assembly, chromosome alignment and microtubule stability in EGBE-exposed oocytes. **(A)** Representative images of spindle morphology and chromosome alignment in control, EGBE-exposed, and NMN-supplemented oocytes. Scale bar, 5 μm. **(B)** The proportion of disorganized spindles was calculated in control, EGBE-exposed, and NMN-supplemented oocytes. **(C)** The proportion of misaligned chromosomes was calculated in control, EGBE-exposed, and NMN-supplemented oocytes. **(D)** Representative images of acetylated α-tubulin in control, EGBE-exposed, and NMN-supplemented oocytes. Scale bar, 5 μm. **(E)** The fluorescence intensity of acetylated α-tubulin was quantified in control, EGBE-exposed, and NMN-supplemented oocytes. **(F)** The acetylation level of α-tubulin was examined in control, EGBE-exposed and NMN-supplemented oocytes by immunoblotting analysis. Data of **(B,C,E)** were expressed as mean percentage (mean ± SEM) of at least three independent experiments. **P < 0.01, ***P < 0.001.

Given that oocyte maturational arrest is usually correlated with the disruption of spindle assembly, we then tested this in EGBE-exposed oocytes by staining them with α-tubulin-FITC antibody. The immunofluorescence results in Figure 2A displayed that most of oocytes in the control exhibited a typical barrel-shaped spindle apparatus with well-aligned chromosomes. By contrast, EGBE-exposed oocytes showed various disorganized spindle morphologies with misaligned chromosomes (Figure 2A). Conversely, NMN supplementation reduced the incidence of aberrant spindle/chromosome structure induced by EGBE exposure (disorganized spindle: 17.2 ± 2.5%, *n* = 37, control, *P* < 0.001 vs. 41.0 ± 2.1%, *n* = 36, EGBE vs. 26.9 ± 2.6%, *n* = 38, NMN, *P* < 0.01; misaligned chromosome: 12.1 ± 1.5%, *n* = 37 control, *P* < 0.001 vs. 67.1 ± 2.1%, *n* = 36, EGBE vs. 34.7 ± 2.8%, *n* = 38, NMN, *P* < 0.01; Figures 2A–C).

One of the most important indicators for the microtubule stability is the acetylation level of α-tubulin in both somatic and germ cells (Montagnac et al., 2013; Xu et al., 2017). Thus, we asked whether the defective spindle organization in EGBE-exposed oocytes results from the perturbed microtubule dynamics through evaluating the acetyl-α-tubulin level. As assessed in Figure 2D, the acetyl-α-tubulin signals were dramatically disappeared in EGBE-exposed oocytes compared to the controls. The quantification of fluorescence intensity and immunoblotting analysis further verified this observation (Figures 2E,F). On the contrary, NMN supplementation significantly increased the acetyl-α-tubulin signals in EGBE-exposed oocytes (37.7 ± 2.9, *n* = 31, control, *P* < 0.01 vs. 12.4 ± 2.7, *n* = 29, EGBE vs. 28.1 ± 2.1, *n* = 30, NMN, *P* < 0.01; Figures 2D–F), suggesting that NMN is able to restore the abnormal spindle formation caused by EGBE exposure by maintaining the microtubule stability.

### NMN Rescues the Actin Dynamics in EGBE-Exposed Porcine Oocytes

**FIGURE 3:**
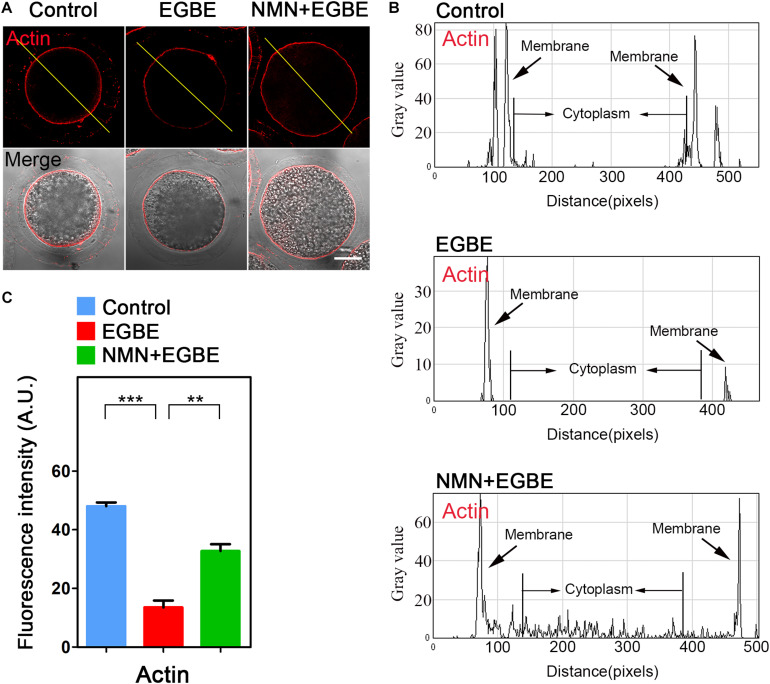
Effect of NMN supplementation on the actin dynamics in EGBE-exposed oocytes. **(A)** Representative images of actin signals in control, EGBE-exposed and NMN-supplemented oocytes. Porcine oocytes were stained with phalloidin-TRITC to show actin filaments. Scale bar, 25 μm. **(B)** The graphs showed the fluorescence intensity profiling of actin filaments in control, EGBE-exposed, and NMN-supplemented oocytes. Pixel intensities were measured along the lines which were drawn across the oocytes. **(C)** The fluorescence intensity of actin filaments on the membrane was quantified in control, EGBE-exposed, and NMN-supplemented oocytes. Data were expressed as mean percentage (mean ± SEM) of at least three independent experiments. **P < 0.01, ***P < 0.001.

In normal oocytes, the actin cytoskeleton takes critical parts in the establishment of cortical polarization and asymmetric spindle positioning. To test whether the actin dynamics is involved in the EGBE effect on oocyte quality, we applied phalloidin-TRITC to display the actin filaments. The data as judged by fluorescence imaging and quantification in Figure 3 validated the impairment of actin integrity in EGBE-exposed oocytes, showing the remarkable decrease of signals on the plasma membrane in comparison with the controls (Figures 3A–C). While, NMN supplementation substantially raised the actin signals in EGBE-exposed oocytes (48.1 ± 1.3, *n* = 29, control, *P* < 0.001 vs. 13.6 ± 1.2, *n* = 31, EGBE vs. 32.7 ± 1.3, *n* = 33, NMN, *P* < 0.01; Figures 3A–C). Overall, all above results illustrate that NMN improves the oocyte maturation via maintaining the integrity of cytoskeleton.

### NMN Improves the Mitochondrial Function in EGBE-Exposed Porcine Oocytes

**FIGURE 4:**
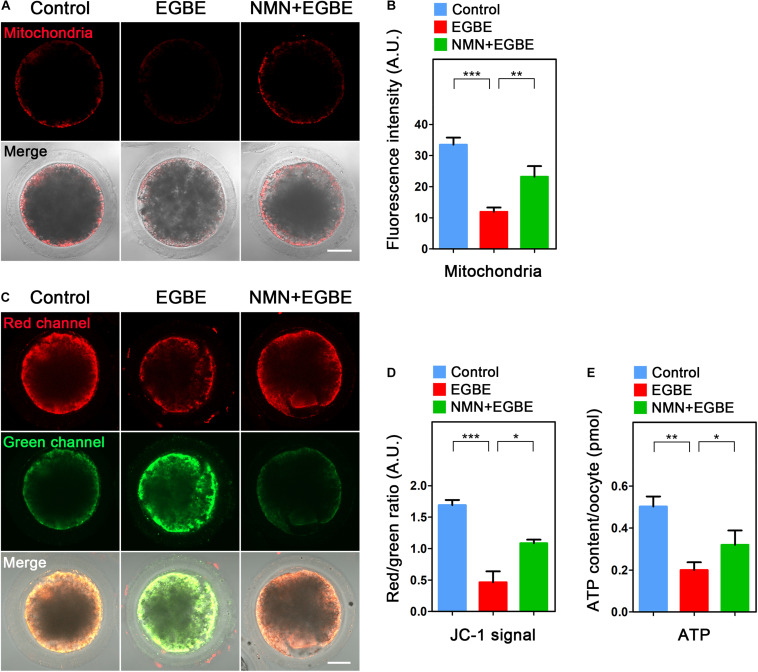
Effect of NMN supplementation on the mitochondrial localization and function in EGBE-exposed oocytes. **(A)** Representative images of mitochondrial distribution in control, EGBE-exposed and NMN-supplemented oocytes. Scale bar, 25 μm. **(B)** The fluorescence intensity of mitochondrial signals was quantified in control, EGBE-exposed, and NMN-supplemented oocytes. **(C)** Mitochondrial membrane potential (ΔΨm) was assessed by JC-1 staining in control, EGBE-exposed and NMN-supplemented oocytes (Red, high ΔΨm; Green, low ΔΨm). Scale bar, 25 μm. **(D)** The ratio of red to green fluorescence intensity was quantified in control, EGBE-exposed, and NMN-supplemented oocytes. **(E)** ATP levels were measured in control, EGBE-exposed and NMN-supplemented oocytes. Data were expressed as mean percentage (mean ± SEM) of at least three independent experiments. *P < 0.05, **P < 0.01, ***P < 0.001.

As an energy factory of cells, mitochondria produce enough ATP for various cellular events, including the cytoskeleton dynamics. We therefore examined if mitochondrial function is disturbed in EGBE-exposed oocytes. Firstly we examined the mitochondrial localization by MitoTracker Red staining and found that a majority of mitochondria distributed around lipid droplets in the subcortex of control porcine oocytes, but lost this normal distribution pattern in EGBE-exposed oocytes (Figure 4A). The quantification of fluorescence intensity revealed that mitochondrial signals dramatically declined in the EGBE-exposed oocytes compared to the controls (Figure 4B). On the contrary, this abnormality was protected by the treatment with NMN (33.5 ± 2.3, *n* = 27, control, *P* < 0.001 vs. 11.9 ± 2.3, *n* = 25, EGBE vs. 23.3 ± 2.4, *n* = 28, NMN, *P* < 0.01; Figures 4A,B). We also applied JC-1 staining to evaluate the mitochondrial membrane potential (ΔΨm). A red fluorescence represented the high membrane potential while a green fluorescence indicated the low membrane potential (Figure 4C). The quantification results displayed that the ratio of red to green signals was considerably decreased in EGBE-exposed oocytes compared to the controls, but elevated in NMN-supplemented oocytes (1.69 ± 0.08, *n* = 29, control, *P* < 0.001 vs. 0.46 ± 0.17, *n* = 31, EGBE vs. 1.09 ± 0.06, *n* = 28, NMN, *P* < 0.05; Figures 4C,D). As production of ATP is the main function of mitochondria, we lastly quantified the ATP levels in three groups of oocytes. The data revealed that ATP content was prominently reduced in EGBE-exposed oocytes in comparison with the controls, but restored after supplementation of NMN (0.5 ± 0.048, *n* = 120, control, *P* < 0.01 vs. 0.2 ± 0.037, *n* = 120, EGBE vs. 0.32 ± 0.068, *n* = 120, NMN, *P* < 0.05; Figure 4E). Altogether, these observations suggest that NMN supplementation improves the EGBE-induced mitochondrial dysfunction in oocytes.

### NMN Removes the Accumulated ROS to Suppress DNA Damage and Apoptosis in EGBE-Exposed Porcine Oocytes

**FIGURE 5:**
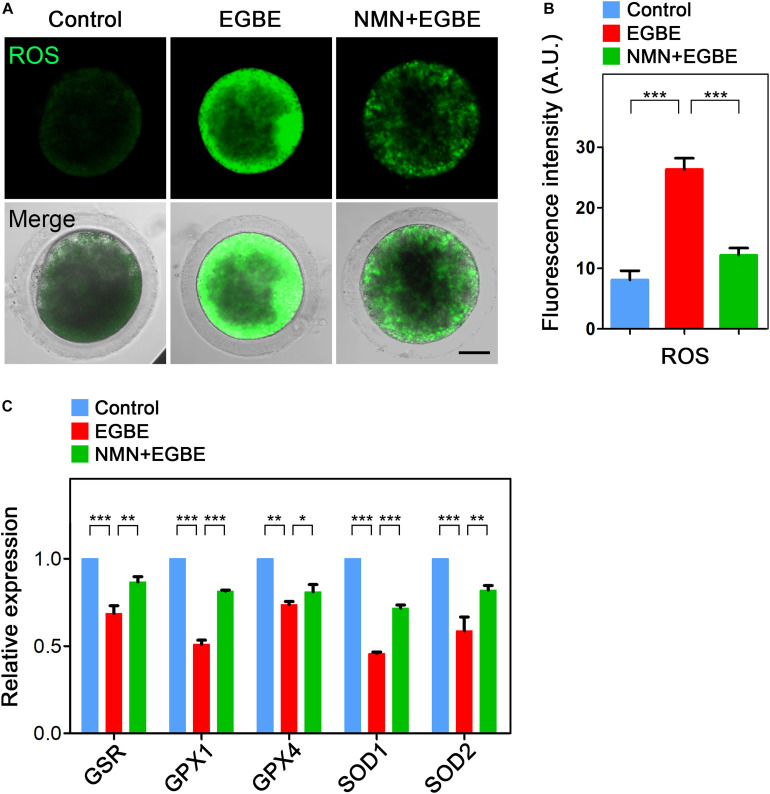
Effect of NMN supplementation on the ROS levels in EGBE-exposed oocytes. **(A)** Representative images of ROS signals in control, EGBE-exposed, and NMN-supplemented oocytes. Scale bar, 20 μm. **(B)** The fluorescence intensity of ROS was quantified in control, EGBE-exposed and NMN-supplemented oocytes. **(C)** Expression of genes related to the anti-oxidation pathway was examined in control, EGBE-exposed, and NMN-supplemented oocytes. Data of **(B,C)** were expressed as mean percentage (mean ± SEM) of at least three independent experiments. *P < 0.05, **P < 0.01, ***P < 0.001.

**FIGURE 6:**
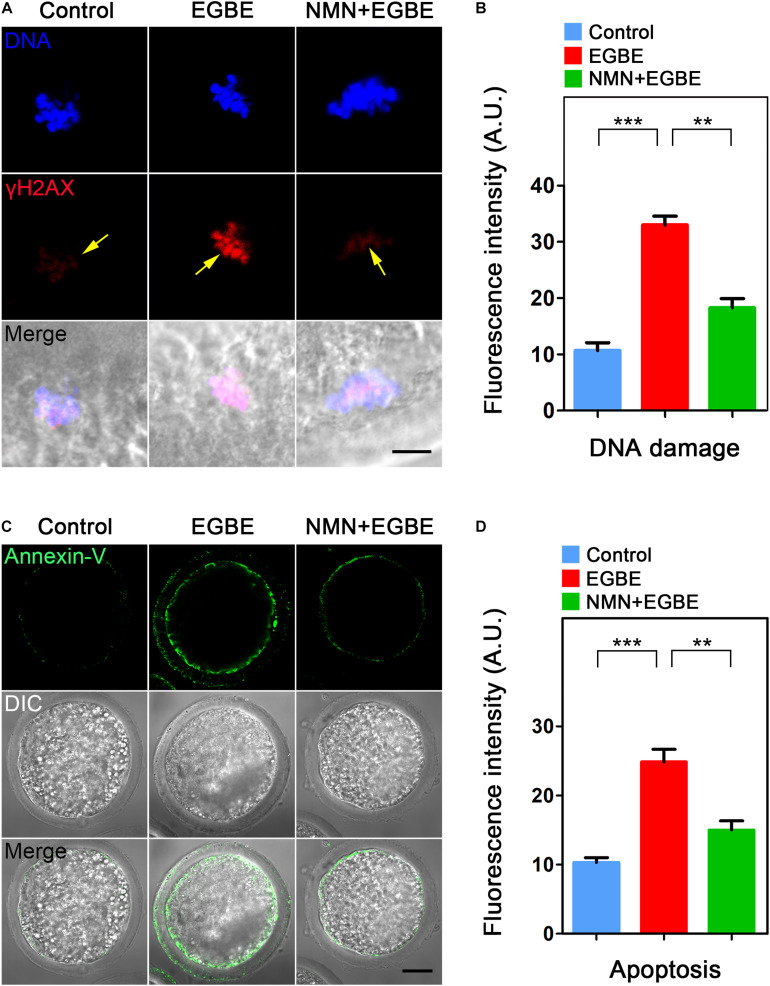
Effect of NMN supplementation on the DNA damage and apoptosis in EGBE-exposed oocytes. **(A)** Representative images of DNA damage in control, EGBE-exposed and NMN-supplemented oocytes. Scale bar, 5 μm. **(B)** The fluorescence intensity of γH2A.X signals was quantified in control, EGBE-exposed, and NMN-supplemented oocytes. **(C)** Representative images of apoptotic oocytes were shown in control, EGBE-exposed and NMN-supplemented groups. Scale bar, 20 μm. **(D)** The proportion of early apoptosis was calculated in control, EGBE-exposed and NMN-supplemented oocytes. Data of **(B,D)** were expressed as mean percentage (mean ± SEM) of at least three independent experiments. **P < 0.01, ***P < 0.001.

Mitochondrial dysfunction is usually correlated with the excessive ROS accumulation, which can result in the oxidative damage to macromolecules in cells and thus induce the apoptosis. To verify this assumption in the context of EGBE exposure, we performed DCFH staining to assess the ROS levels in control and EGBE-exposed oocytes. The data revealed that ROS signals were hardly detected in the cytoplasm of control oocytes, but considerably elevated in EGBE-exposed oocytes (Figure 5A). Expectedly, NMN supplementation markedly lowered the ROS signals in EGBE-exposed oocytes (8.1 ± 1.5, *n* = 23, control, *P* < 0.001 vs. 26.3 ± 1.9, *n* = 24, EGBE vs. 12.2 ± 1.2, *n* = 23, NMN, *P* < 0.001; Figures 5A,B). In addition, we validated the downregulated expression of a couple of genes implicated in the anti-oxidation pathways in EGBE-exposed oocytes, but recovered in NMN-supplemented oocytes (Figure 5C). As the high level of ROS usually induces DNA damage and apoptosis (Ratan et al., 1994; Ozben, 2007), we next assessed DNA damage accumulation by γH2A.X antibody staining and early apoptosis occurrence by Annexin-V staining. The fluorescent images and intensity measurement analysis displayed that γH2A.X signals were enhanced on the chromosomes in EGBE-exposed oocytes compared to the controls (Figures 6A,B). Whereas supplementation with NMN significantly reduced the accumulation of DNA damage (10.7 ± 1.4, *n* = 21, control, *P* < 0.001 vs. 33.0 ± 1.6, *n* = 23, EGBE vs. 18.3 ± 1.6, *n* = 21, NMN, *P* < 0.01; Figures 6A,B). Furthermore, we observed that Annexin-V signals were hardly found in control oocytes, but robustly present on the membrane of EGBE-exposed oocytes (Figure 6C). Concordantly, the frequency of apoptosis was significantly elevated in EGBE-exposed group, but decreased in NMN-supplemented group (10.3 ± 0.7, *n* = 29, control, *P* < 0.001 vs. 24.9 ± 1.8, *n* = 31, EGBE vs. 15.1 ± 1.3, *n* = 27, NMN, *P* < 0.01; Figures 6C,D). Collectively, our findings demonstrate that NMN.

## Discussion

Ethylene glycol butyl ether (EGBE), which belongs to the chemical family of glycol ethers, is a commonly used solvent for hydraulic fluids, varnishes and resins, and a component of various household products such as floor polishes and waxes, as well as cleaning compositions for upholstery, leather and glass (Rambourg-Schepens et al., 1988; Browning and Curry, 1994). In recent years, an increasing amount of studies have reported that compounds in the glycol ethers have adverse impact on the reproductive system. Ethylene glycol monoethyl ether (EGEE) severely reduces the activities of glutathione (GSH), superoxide dismutase (SOD), and catalase (CAT), and thus destroys the redox balance in the rat testes and sperm (Adedara and Farombi, 2010). The percentage of tetraploid sperm in male rats exposed to EGEE for 14 days (400 mg/kg/day) was significantly higher than that in controls (Yu et al., 1999). Ethylene glycol monomethyl ether (EGME) has been shown to induce the apoptosis of mouse oocytes and granulosa cells (Weng et al., 2010). Supplementation of EGME during oocyte development also affected nuclear maturation and impaired the meiotic process of Xenopus laevis eggs (Fort et al., 2002). However, how EGBE affects the quality of female germ cells remains to be further explored.

To this end, we investigated the extrusion of polar body and the expansion of cumulus cells, two key indicators for the normal porcine oocyte maturation. EGBE-exposed oocytes displayed a reduced frequency of polar body extrusion with weakened expansion of cumulus cells in a dose-dependent manner, suggesting that EGBE exposure leads to the failure of oocyte meiotic maturation. Since the impairment of cytoskeletal assembly often results in the defect of cell division in both meiotic and mitotic cells (Azoury et al., 2008; Heng and Koh, 2010), we further explored the negative effect of EGBE exposure on the oocyte meiotic progression by testing the cytoskeleton dynamics. Our data showed that EGBE exposure disrupted the spindle organization through compromising the stability of microtubules, which was coupled with the chromosome misalignment. Moreover, EGBE exposure disturbed the integrity of actin, another essential component of cytoskeleton, on the oocyte membrane. Thus, these findings demonstrate that the perturbed oocyte maturation induced by EGBE exposure is attributed to the defective cytoskeleton dynamics.

Mitochondrion functions as a primary factory in oocytes to generate ATP source for cell development (Dumollard et al., 2007; Niu et al., 2019), and its distribution pattern has been considered as one of the critical indicators for evaluating the oocyte cytoplasmic maturation. Our findings revealed that the localization, membrane potential, and ATP production of mitochondria were disturbed in EGBE-exposed oocytes, implying that EGBE exposure impairs the oocyte cytoplasmic maturation by destructing the mitochondrial distribution and function. Consequently, ROS levels were increased to induce the accumulation of DNA damage and occurrence of apoptosis in EGBE-exposed oocytes.

It has been reported that NMN, a key NAD^+^ intermediate, recovers the function of cerebromicrovascular endothelial and neurovascular coupling responses in aged mice by attenuating mitochondrial production of ROS (Tarantini et al., 2019). In addition, we recently evidenced that *in vivo* supplementation of NMN restores the NAD^+^ level to ameliorate the quality of oocytes from aged mice *via* maintaining the chromosome euploidy and fertilization ability (Miao et al., 2020). In line with this protective effect NMN, we validated that NMN could recover the EGBE exposure-induced meiotic defects by restoring NAD^+^ level and removing excessive ROS, hence suppressing DNA damage accumulation and apoptosis occurrence in porcine oocytes.

**FIGURE 7:**
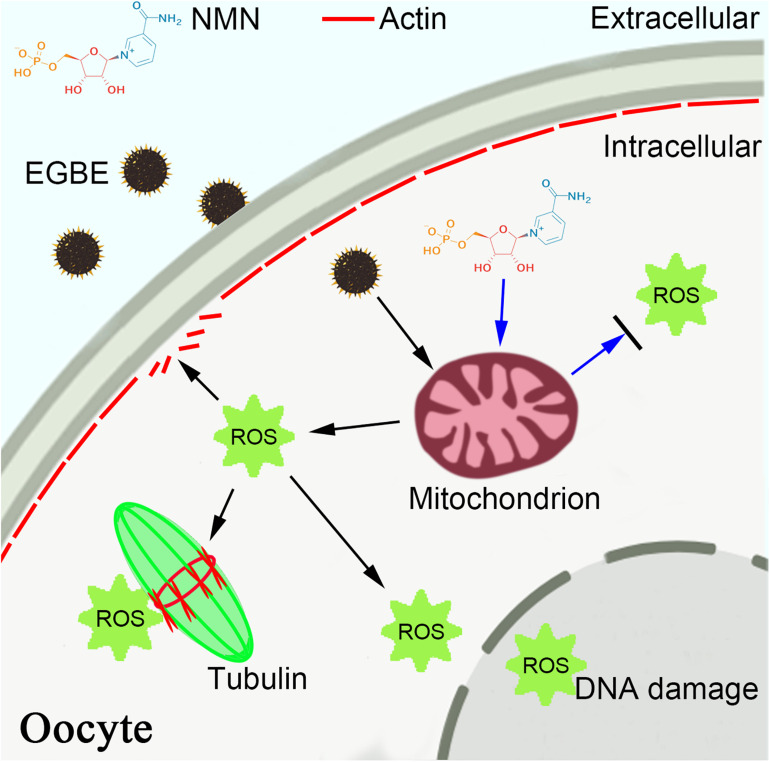
Schematic diagram of NMN effect on the EGBE-exposed oocytes. EGBE exposure leads to the aberrant spindle assembly, chromosome alignment, and actin polymerization during porcine oocyte meiotic maturation by impairing the mitochondrial functions. NMN supplementation restores the meiotic defects induced by EGBE exposure through improving the mitochondrial function and eliminating the accumulation of ROS, thereby inhibiting the occurrence of DNA damage and apoptosis.

To sum up, we provide a body of evidence documenting that NMN protects the oocytes from EGBE exposure by restoring the mitochondrial function and suppressing EGBE-induced production of ROS and apoptosis (Figure 7).

## Data Availability Statement

The original contributions presented in the study are included in the article/supplementary material, further inquiries can be directed to the corresponding author/s.

## Ethics Statement

The animal study was reviewed and approved by the Animal Research Institute Committee of Nanjing Agricultural University, China.

## Author Contributions

BX and YF designed the research. YM, XL, XS, QG, JC, and RW performed the experiments. YM, XL, YF, and BX analyzed the data. YM, YF, and BX wrote the manuscript. All authors contributed to the article and approved the submitted version.

## Conflict of Interest

The authors declare that the research was conducted in the absence of any commercial or financial relationships that could be construed as a potential conflict of interest.

## References

[B1] Adedara I. A. Farombi E. O. (2010). Induction of oxidative damage in the testes and spermatozoa and hematotoxicity in rats exposed to multiple doses of ethylene glycol monoethyl ether. *Hum. Exp. Toxicol.* 29 801–812. 10.1177/0960327109360115 20172899

[B2] Anastasiou D. Krek W. (2006). SIRT1: linking adaptive cellular responses to aging-associated changes in organismal physiology. *Physiology* 21 404–410. 10.1152/physiol.00031.2006 17119153

[B3] Azoury J. Lee K. W. Georget V. Rassinier P. Leader B. Verlhac M. H. (2008). Spindle positioning in mouse oocytes relies on a dynamic meshwork of actin filaments. *Curr. Biol.* 18 1514–1519. 10.1016/j.cub.2008.08.044 18848445

[B4] Bauer P. Weber M. Mur J. M. Protois J. C. Bollaert P. E. Condi A. (1992). Transient non-cardiogenic pulmonary edema following massive ingestion of ethylene glycol butyl ether. *Intensive Care Med.* 18 250–251. 10.1007/BF01709843 1430593

[B5] Browning R. G. Curry S. C. (1994). Clinical toxicology of ethylene glycol monoalkyl ethers. *Hum. Exp. Toxicol.* 13 325–335. 10.1177/096032719401300508 8043314

[B6] Camacho-Pereira J. Tarrago M. G. Chini C. C. S. Nin V. Escande C. Warner G. M. (2016). CD38 dictates age-related NAD decline and mitochondrial dysfunction through an SIRT3-dependent mechanism. *Cell Metab.* 23 1127–1139. 10.1016/j.cmet.2016.05.006 27304511 PMC4911708

[B7] Cheever K. L. Plotnick H. B. Richards D. E. Weigel W. W. (1984). Metabolism and excretion of 2-ethoxyethanol in the adult male rat. *Environ. Health Perspect.* 57 241–248. 10.1289/ehp.8457241 6437805 PMC1568278

[B8] Cicolella A. (2006). [Glycol ethers reproductive risks]. *Gynecol. Obstet. Fertil.* 34 955–963. 10.1016/j.gyobfe.2006.07.034 16987687

[B9] Combelles C. M. Albertini D. F. (2003). Assessment of oocyte quality following repeated gonadotropin stimulation in the mouse. *Biol. Reprod.* 68 812–821. 10.1095/biolreprod.102.008656 12604630

[B10] Croteau D. L. Fang E. F. Nilsen H. Bohr V. A. (2017). NAD(+) in DNA repair and mitochondrial maintenance. *Cell Cycle* 16 491–492. 10.1080/15384101.2017.1285631 28145802 PMC5384578

[B11] Csiszar A. Labinskyy N. Jimenez R. Pinto J. T. Ballabh P. Losonczy G. (2009a). Anti-oxidative and anti-inflammatory vasoprotective effects of caloric restriction in aging: role of circulating factors and SIRT1. *Mech. Ageing Dev.* 130 518–527. 10.1016/j.mad.2009.06.004 19549533 PMC2756526

[B12] Csiszar A. Labinskyy N. Pinto J. T. Ballabh P. Zhang H. Losonczy G. (2009b). Resveratrol induces mitochondrial biogenesis in endothelial cells. *Am. J. Physiol. Heart Circ. Physiol.* 297 H13–H20. 10.1152/ajpheart.00368.2009 19429820 PMC2711732

[B13] Csiszar A. Labinskyy N. Podlutsky A. Kaminski P. M. Wolin M. S. Zhang C. (2008). Vasoprotective effects of resveratrol and SIRT1: attenuation of cigarette smoke-induced oxidative stress and proinflammatory phenotypic alterations. *Am. J. Physiol. Heart Circ. Physiol.* 294 H2721–H2735. 10.1152/ajpheart.00235.2008 18424637 PMC2551743

[B14] Das A. Huang G. X. Bonkowski M. S. Longchamp A. Li C. Schultz M. B. (2018). Impairment of an endothelial NAD(+)-H2S signaling network is a reversible cause of vascular aging. *Cell* 173:e20. 10.1016/j.cell.2018.02.008 29570999 PMC5884172

[B15] Dumollard R. Duchen M. Carroll J. (2007). The role of mitochondrial function in the oocyte and embryo. *Curr. Top. Dev. Biol.* 77 21–49. 10.1016/S0070-2153(06)77002-8 17222699

[B16] Fort D. J. Mathis M. B. Guiney P. D. Weeks J. A. (2018). Inhibition of germinal vesicle breakdown in Xenopus oocytes in vitro by a series of substituted glycol ethers. *J. Appl. Toxicol.* 38 628–637. 10.1002/jat.3567 29205417

[B17] Fort D. J. Mclaughlin D. W. Rogers R. L. Buzzard B. O. (2002). Effect of endocrine disrupting chemicals on germinal vesicle breakdown in *Xenopus* in vitro. *Drug Chem. Toxicol.* 25 293–308. 10.1081/dct-120005892 12173250

[B18] Heng Y. W. Koh C. G. (2010). Actin cytoskeleton dynamics and the cell division cycle. *Int. J. Biochem. Cell Biol.* 42 1622–1633. 10.1016/j.biocel.2010.04.007 20412868

[B19] Hsieh G. Y. Wang J. D. Cheng T. J. Chen P. C. (2005). Prolonged menstrual cycles in female workers exposed to ethylene glycol ethers in the semiconductor manufacturing industry. *Occup. Environ. Med.* 62 510–516. 10.1136/oem.2004.016014 16046602 PMC1741062

[B20] Kennedy C. H. Bechtold W. E. Chang I. Y. Henderson R. F. (1993). Effect of dose on the disposition of 2-ethoxyethanol after inhalation by F344/N rats. *Fundam. Appl. Toxicol.* 21 486–491. 10.1006/faat.1993.1124 8253301

[B21] Melnick R. L. (1984). Toxicities of ethylene glycol and ethylene glycol monoethyl ether in Fischer 344/N rats and B6C3F1 mice. *Environ. Health Perspect.* 57 147–155. 10.1289/ehp.8457147 6499799 PMC1568296

[B22] Miao Y. Cui Z. Gao Q. Rui R. Xiong B. (2020). Nicotinamide mononucleotide supplementation reverses the declining quality of maternally aged oocytes. *Cell Rep.* 32:107987. 10.1016/j.celrep.2020.107987 32755581

[B23] Montagnac G. Meas-Yedid V. Irondelle M. Castro-Castro A. Franco M. Shida T. (2013). alphaTAT1 catalyses microtubule acetylation at clathrin-coated pits. *Nature* 502 567–570. 10.1038/nature12571 24097348 PMC3970258

[B24] Multigner L. Catala M. Cordier S. Delaforge M. Fenaux P. Garnier R. (2005). The INSERM expert review on glycol ethers: findings and recommendations. *Toxicol. Lett.* 156 29–37. 10.1016/j.toxlet.2003.12.077 15705485

[B25] Niu Y. J. Nie Z. W. Shin K. T. Zhou W. Cui X. S. (2019). PINK1 regulates mitochondrial morphology via promoting mitochondrial fission in porcine preimplantation embryos. *FASEB J.* 33 7882–7895. 10.1096/fj.201802473R 30897005

[B26] Ozben T. (2007). Oxidative stress and apoptosis: impact on cancer therapy. *J. Pharm. Sci.* 96 2181–2196. 10.1002/jps.20874 17593552

[B27] Pantazi E. Zaouali M. A. Bejaoui M. Folch-Puy E. Ben Abdennebi H. Varela A. T. (2015). Sirtuin 1 in rat orthotopic liver transplantation: an IGL-1 preservation solution approach. *World J. Gastroenterol.* 21 1765–1774. 10.3748/wjg.v21.i6.1765 25684941 PMC4323452

[B28] Rambourg-Schepens M. O. Buffet M. Bertault R. Jaussaud M. Journe B. Fay R. (1988). Severe ethylene glycol butyl ether poisoning. Kinetics and metabolic pattern. *Hum. Toxicol.* 7 187–189. 10.1177/096032718800700215 3378807

[B29] Ratan R. R. Murphy T. H. Baraban J. M. (1994). Macromolecular synthesis inhibitors prevent oxidative stress-induced apoptosis in embryonic cortical neurons by shunting cysteine from protein synthesis to glutathione. *J. Neurosci.* 14 4385–4392. 8027786 10.1523/JNEUROSCI.14-07-04385.1994 PMC6577015

[B30] Scofield E. H. Henderson W. M. Funk A. B. Anderson G. L. Smith M. A. (2006). Diethylene glycol monomethyl ether, ethylene glycol monomethyl ether and the metabolite, 2-methoxyacetic acid affect in vitro chondrogenesis. *Reprod. Toxicol.* 22 718–724. 10.1016/j.reprotox.2006.05.005 16829022

[B31] Tarantini S. Valcarcel-Ares M. N. Toth P. Yabluchanskiy A. Tucsek Z. Kiss T. (2019). Nicotinamide mononucleotide (NMN) supplementation rescues cerebromicrovascular endothelial function and neurovascular coupling responses and improves cognitive function in aged mice. *Redox Biol.* 24:101192. 10.1016/j.redox.2019.101192 31015147 PMC6477631

[B32] Wang R. S. Ohtani K. Suda M. Kitagawa K. Nakayama K. Kawamoto T. (2007). Reproductive toxicity of ethylene glycol monoethyl ether in Aldh2 knockout mice. *Ind. Health* 45 574–578. 10.2486/indhealth.45.574 17878629

[B33] Wang R. S. Ohtani K. Suda M. Nakajima T. (2006). Inhibitory effect of ethylene glycol monoethyl ether on rat sperm motion. *Ind. Health* 44 665–668. 10.2486/indhealth.44.665 17085930

[B34] Welsch F. (2005). The mechanism of ethylene glycol ether reproductive and developmental toxicity and evidence for adverse effects in humans. *Toxicol. Lett.* 156 13–28. 10.1016/j.toxlet.2003.08.010 15705484

[B35] Weng S. P. Wu T. C. Chen S. U. Wu J. Lin C. C. Yang Y. C. (2010). The impact of ethylene glycol monomethyl ether on ovarian function may extend to the next generation in female mice: a preliminary study. *Reprod. Toxicol.* 29 452–457. 10.1016/j.reprotox.2010.03.009 20380876

[B36] Xu Z. Schaedel L. Portran D. Aguilar A. Gaillard J. Marinkovich M. P. (2017). Microtubules acquire resistance from mechanical breakage through intralumenal acetylation. *Science* 356 328–332. 10.1126/science.aai8764 28428427 PMC5457157

[B37] Yoshino J. Mills K. F. Yoon M. J. Imai S. (2011). Nicotinamide mononucleotide, a key NAD(+) intermediate, treats the pathophysiology of diet- and age-induced diabetes in mice. *Cell Metab.* 14 528–536. 10.1016/j.cmet.2011.08.014 21982712 PMC3204926

[B38] Yu I. J. Lee J. Y. Chung Y. H. Kim K. J. Han J. H. Cha G. Y. (1999). Co-administration of toluene and xylene antagonized the testicular toxicity but not the hematopoietic toxicity caused by ethylene glycol monoethyl ether in Sprague-Dawley rats. *Toxicol. Lett.* 109 11–20. 10.1016/s0378-4274(99)00063-6 10514026

